# Successful treatment of a unique chronic multi-bacterial scalp infection with *N*-chlorotaurine, *N*-bromotaurine and bromamine T

**DOI:** 10.1099/acmi.0.000126

**Published:** 2020-04-24

**Authors:** Anthony M. Kyriakopoulos, Markus Nagl, Dorothea Orth-Höller, Janusz Marcinkiewicz, Stella Baliou, Vassilis Zoumbourlis

**Affiliations:** ^1^​ Nasco AD Biotechnology Laboratory, 11 Sachtouri Str., Piraeus 18536, Greece; ^2^​ Department of Hygiene, Microbiology and Social Medicine, Institute of Hygiene and Medical Microbiology, Medical University of Innsbruck, Innsbruck, Austria; ^3^​ Department of Immunology, Jagiellonian University Medical College, Krakow, Poland; ^4^​ National Hellenic Research Foundation, 48 Vasileos Konstantinou Str., Athens, Greece

**Keywords:** chronic multi-bacterial skin infection, *N*-chlorotaurine, *N*-bromotaurine, bromamine T

## Abstract

Microbial species can act in synergy to circumvent environmental stress conditions and survive. In addition, biofilms are a serious public-health issue globally and constitute a clinical emergency. Infection persistence, increased morbidity and mortality, and antibiotic resistance are consequences of poly-microbial synergy. Due to inherited complexity and synergy between numerous species, newer antimicrobial agents of increased efficacy and tolerability are needed. In this unique medical case, a chronic (9 year) multi-bacterial scalp infection was differentially diagnosed from other inflammatory skin disorders by prolonged microbiological culture. The bacterial species found seem to have caused lesions of visible biofilm not documented previously in the medical literature. This complicated infection was treated successfully and rapidly with the combined topical application of the active halogen compounds *N*-chlorotaurine, *N*-bromotaurine and bromamine T, which is in contrast to the previous failed systemic and topical therapeutic approaches. This study strengthens the case for the use of active halogen compounds against multi-bacterial infections of the skin in the future, without the occurrence of resistance.

## Introduction

Microbial species naturally evolve and optimize mechanisms to resist killing by harmful conditions, such as the presence of antibiotics. These mechanisms create ‘*un s'en vient*’ echoes of the resulting antimicrobial therapy failures that eventually compromise the practice of advanced medical microbiology. Poly-microbial synergy is clinically important [1]. The production of biofilms due to microbial synergy elevates patient mortality and morbidity [2–5]. Complex sepsis infections with coagulase-negative staphylococci are linked to persistent colonization after antibiotic treatment on superficial sites, like umbilical catheters [2]. Increased mortality from initial infection is higher with biofilm-producing strains as compared to non-biofilm-producing strains [3]. Such infections may be complemented by frequent synergistic infections due to *Candida* species, leading to severe clinical complications [4]. Fifty thousand deaths per year in the USA alone result from persistent *Candida* species infections due to the production of biofilms [4]. Severe biofilm infections caused by *Staphylococcus aureus* and *Enterobacteriaceae* are also a significant issue [5].

Mixed aerobic and anaerobic bacteria species comprise poly-microbial synergistic infections and provide nutritionally distinct microenvironments for the survival of the different species during the infection [6–8]. Clinical investigations suggest that synergizing biofilm-producing bacterial strains account for a 300-fold elevated patient mortality as compared to strains that are do not produce a biofilm [9]. Microbial analysis of samples from chronic wounds also suggests that persistence of infection is tightly associated with synergizing bacteria, complicating the healing process [10–13].

The overwhelming resistance to antimicrobial agents due to bacterial synergy during infection, which is also seen for biofilm-forming bacteria, requires novel approaches to ensure successful treatment [14–16]. One not novel, but frequently underestimated, approach is to apply antiseptics instead of antibiotics, if topical treatment is possible. In this regard, novel substances with high tissue tolerability have become of interest, for instance active halogen compounds. Haloamines show increased antimicrobial activity compared to antibiotics, and due to their oxidizing properties the microbes have a negligible chance to develop resistance [17, 18]. *N*-Chlorotaurine (NCT) and *N*-bromotaurine (NBrT) are natural representatives of this type of compound. The tolerability and efficacy of NCT for topical treatment of infections of different body sites has been demonstrated in clinical studies and single cases, for instance on the skin, in the eye, in the ear–nose–throat region and in the urinary bladder amongst others [17, 18]. NBrT has shown tolerability and efficacy in the treatment of acne [18]. Recently, NCT and NBrT have been also combined as an approach to potentiate their properties. This was a favourable therapeutic choice in the case of a herpes zoster infection refractory to common antivirals [19], and truly life-saving in the case of a multiresistant *S. aureus* chronic wound infection [20]. Moreover, both haloamines inhibit bacterial biofilm formation and inactivate biofilm as proved *in vitro* [21, 22], with NBrT showing superiority in this activity [21]. Due to the long-term structural instability of NBrT, however, more stable agents like bromamine T (BAT), with similar anti-inflammatory and antimicrobial activities, have been devised and considered for clinical anti-infective use [22].

In this case report, 9-year-old lesions of the scalp resembling atypical pustular erosions with eschars proved to be an unidentified chronic infection of multiple, synergizing microbial species. From this infectious tissue, seven distinct bacterial species, namely *Pseudomonas fluorescens *, *Staphylococcus capitis, Staphylococcus lugdunensis, Staphylococcus epidermidis*, *Bacillus pumilus *, *Kocuria marina* and *Aerococcus viridans *, were isolated. The infectious masses growing on and within the skin were resistant to previous topical and systemic administrations of multiple antibiotics, disinfectants, cytostatic agents and cryosurgery. Possibly, this was due to biofilm formation. In the end, the infection was successfully treated and rapidly cured with the combined topical application of NCT, NBrT and BAT. Within 25 days, a totally healed skin tissue with complete disappearance of the infectious plaques was achieved.

## Methods

### Microbial culture, identification and resistance testing

Swabs and small fragments from the area of chronic infection were cultured on Columbia blood agar (Becton Dickinson) under aerobic and anaerobic conditions, for 48 days at 37 °C. Bacterial species were identified by matrix-assisted laser desorption/ionization-time of flight mass spectrometry (MALDI-TOF MS; Bruker Daltonics) using the direct smear method. A score above 1.7 was considered valid [23].

Resistance against antibiotics was assessed by disc-diffusion test on Müller–Hinton agar plates, according to European Committee on Antimicrobial Susceptibility Testing standards [24]. For those bacteria where no species-related zone-diameter breakpoints existed, the Etest method with PK-PD (non-species related) breakpoints was used. The following antibiotics were tested: penicillin (not for *Pseudomonas* spp.); ampicillin (not for *Pseudomonas* spp.); ampicillin + clavulanic acid (not for *Pseudomonas* spp. and *Aerococcus* spp.); piperacillin + tazobactam; first-, second-, third- and fourth-generation cephalosporins (not for *Aerococcus* spp. and only partially for *Pseudomonas* spp.); carbapenems; ciprofloxacin and levofloxacin; aminoglycosides (not for *Aerococcus* spp.); vancomycin (not for *Pseudomonas* spp.). For *Staphylococcus* spp., the following additional antibiotics were tested: clindamycin, erythromycin, doxycycline, fosfomycin, cefoxitin and linezolid.

### Preparation of NCT, NBrT and BAT solutions

NCT and NBrT solutions at a concentration of 1.0 % (55 mM NCT, 49 mM NBrT) were freshly prepared in distilled water as described previously in 2016 and 2019 [19, 20]. The solutions were put into sterile spray bottles, which released approximately 130 µl per single puff. BAT crystalline sodium salt was provided by the Institute of Hygiene and Medical Microbiology, Medical University of Innsbruck, Innsbruck, Austria. A 0.1 % (3.2 mM) BAT solution in distilled water was prepared in similar sterile spray bottles as for NCT and NBrT.

### Quantitative *in vitro* killing assays

Tests were done in 0.1 M phosphate buffer at pH 7.1 and 37 °C in a water bath. Separate solutions of 1 % NCT (55 mM), 0.02 % NBrT (0.98 mM) and 0.001 % (32 µM) BAT were prepared in the buffer. Separate overnight cultures of the bacteria at 37 °C in tryptic soy broth were washed twice in saline and diluted to 1×10^8^ c.f.u. ml^−1^ in saline . Bacterial suspensions of 40 µl were added to 3.96 ml of each test solution at time zero and incubated at 37 °C. After different incubation times (1, 5, 15, 30 min), 100 µl aliquots were removed and mixed with 900 µl 1 % methionine/1 % histidine in distilled water to inactivate the antiseptic. Aliquots (50 µl) were plated in duplicate on Columbia blood agar using an automatic spiral plater (model WASP 2; Don Whitley UK), allowing a detection limit of 100 c.f.u. ml^−1^ taking into account both plates. Plates were grown at 37 °C for 48 h, and colonies were counted. Plates with no growth were incubated for a further 5 days. Controls in 0.1 M phosphate buffer without antiseptics were performed and showed no reduction in the number of c.f.u., as was also found for the respective inactivation controls [25].

## Results

### Clinical case

**Fig. 1.**
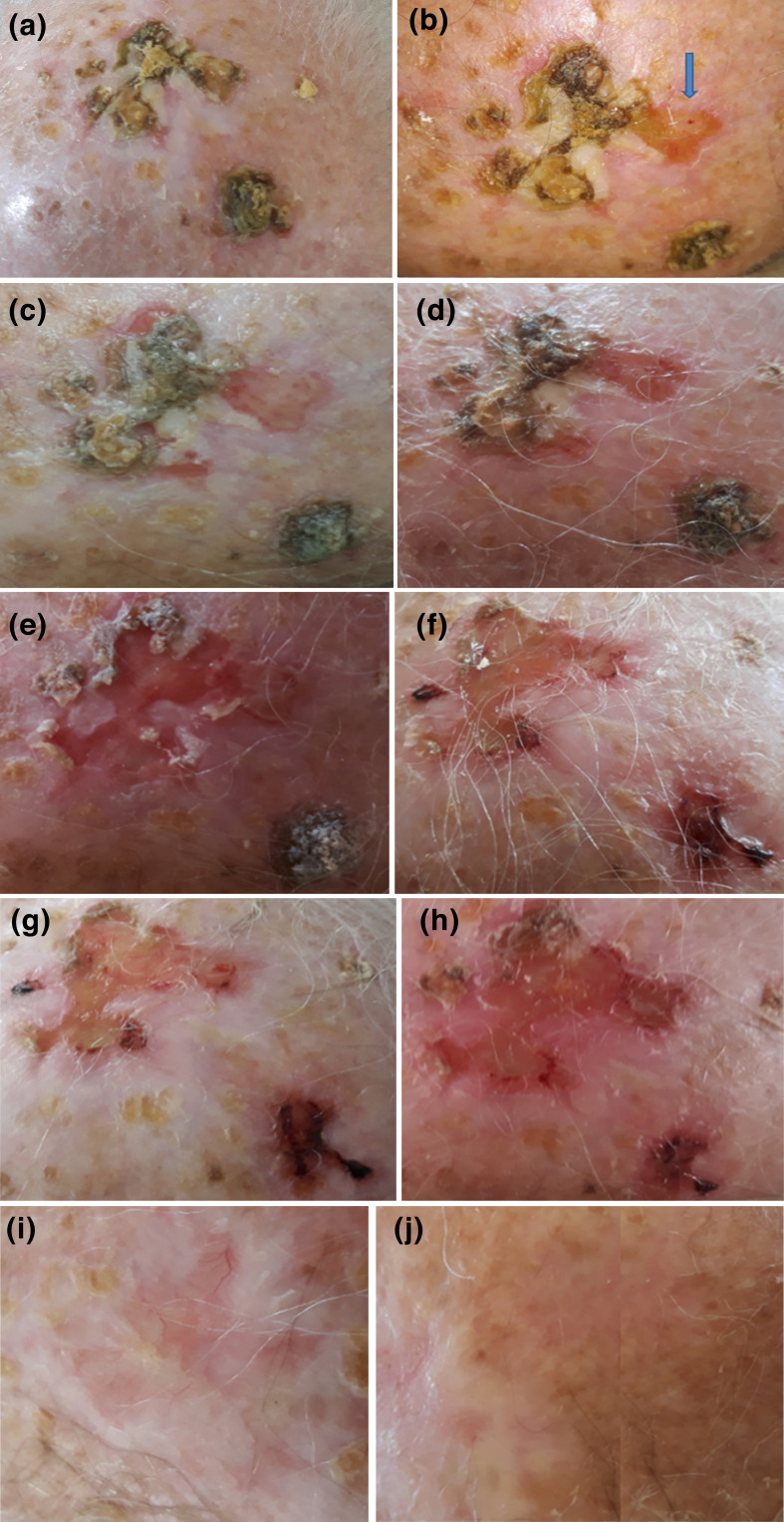
Treatment of the chronic multi-bacterial infection of the scalp over 60 days with a combination of 1 % NCT, 1 % NBrT and 0.1 % BAT. (a) Lesions growing on the elderly patient’s scalp resembled pustular erosions with eschars. (b) Assuming an infectious origin, some lesions were detached successfully (indicated by an arrow) by using a gauze soaked with a 1 % NCT solution. Tissue and swabs were collected and sent for microbial culture. (c) On day 3, treatment with 1 % NCT showed visible but slow regression of the lesions. Combination therapy with 1 % NCT and 1 % NBrT was commenced. (d) The next day (day 4), the 1 % NCT and 1 % NBrT combination treatment had accelerated the regression and softening of the lesions. (e) By the fifth day of treatment, lesions together with a subcutaneous portion of skin could be partly removed without serious bleeding. Application of 0.1 % BAT twice daily was commenced at home. (f) Evident accelerated tissue repair and almost total clearance of remaining lesions. (g) Wounds were epithelialized, with no evident recurrence of lesions. (h) Further epithelialization of the wounds from the previous lesions. (i) Remarkable epithelialization and tissue repair induced the decision to end the treatments at the clinic. (j) The skin was completely epithelialized and returned to normal pigmentation. Application of 0.1 % BAT was stopped.

An 85-year-old woman was examined in Nasco AD Biotech.Lab. affiliated clinic for a set of pustular erosions with eschars growing on her scalp (Fig. 1a). A recent haematology examination had recorded that the patient suffered from mild eosinophilia [26] of 5 900 cells µl^−1^, a slightly low haematocrit level of 33.4 % and 11.1 g haemoglobin dl^−1^. From the extensive medical record taken, there was no coherent diagnosis over the whole 9 years of her disease period. Prior to the formation of the lesions on her scalp, the patient had experienced an accidental fall from a window sill, which resulted in a severe bleeding wound on her scalp that required her hospitalization. The wounded area never healed completely. According to the patient's information, similar lesions to the ones presented to us began to develop after a month of topical therapy with wound dressings subsequent to her dismissal from the hospital. Initial histology from whole biopsy specimens performed at that time (9 years ago) resulted in ulceration and inflammatory abscesses. Numerous multinucleated foreign body giant cells were described. This appears unusual to us, since these are known to develop from immune reactions to biomaterials [27, 28].

A month later, the patient was hospitalized again and treated systemically with a combination of methylprednisone at 1 mg^−1^ kg daily and cyclosporine at 5 mg^−1^ kg daily for 5 days and topically with 2 % fusidic acid dressings, assuming pyoderma gangrenosum. Unfortunately, the treatment was of no benefit and the lesions worsened after a few days. Synoptically, in the following 9 years, the patient was treated repeatedly with local iodine; gentamicin and dexamethasone cream, at 1 mg g^−1^ and 0.5 mg g^−1^, respectively; 2 % fusidic acid and 1 % hydrocortisone; 2 % miconazole and 1 % hydrocortisone; and finally with continuous painful cryosurgeries without any therapeutic effect.

### Microbiological investigation

Upon initial examination of the patient at our clinic, the lesions resembled infectious material. To obtain adequate material for microbiological examination, the lesions were soaked using sterile gauze embedded in 1 % NCT solution until they turned soft. In this way, material from deeper layers could be detached from the scalp avoiding pain or bleeding (Fig. 1b). Small samples from multiple biofilm-like infection areas, as well as swabs, were taken and subjected to aerobic and anaerobic culture on Columbia blood agar. Sequential and prolonged culturing proved our medical assumption by isolating a plethora of bacterial species. The bacterial species isolated and identified by MALDI-TOF MS were: the Gram-positive cocci *S. lugdunensis *, *S. capitis *, *S. epidermidis *, *A. viridans* and *K. marina *; the Gram-positive rod *B. pumilus;* and the Gram-negative rod *P. fluorescens* (Table 1).

**Table 1.** The bacterial species isolated from the chronic infection tissue: their pathogenicity and ability to form biofilms

Bacterial species	Pathogenicity*	Biofilm-forming ability†
* P. fluorescen *s complex [31]	Opportunistic pathogen	Active biofilm formation on human cells
* S. lugdunensis *	Opportunistic pathogen	Active biofilm formation on medical devices [34]
* A. viridans *	Opportunistic invasive pathogen [36]	Not known to produce or be involved in biofilms
* B *. *pumilus *‡	Exceptional pathogen [37-39]	Only known for aquatic environments [41]
* K.* *marina *‡	Emerging opportunistic and nosocomial pathogen [43-44]	Known not to produce biofilms in humans [43]
* S. epidermidis *	Opportunistic and nosocomial pathogen	Biofilm-forming ability on inserted devices [47]
* S. capitis *	Opportunistic and nosocomial pathogen	Biofilm-forming ability on inserted devices under certain conditions [47]

*Strains are pathogenic in wounds, as well as in other systemic infections.

††Ability to form biofilm as a single species on human tissue, as well as on other environmental niches including plants.

‡Different species within the same genus, namely *K. kristinae* [45] or *B. subtilis* [41], are known as active biofilm producers in human infections.

### Treatment with NCT, NBrT and BAT

The lesions on the scalp skin seemed to be due to a synergy of bacteria forming a unique chronic wound infection and possibly a biofilm not previously reported in medical science. Therefore, antimicrobial treatment at our hospital was considered. As the patient refused further systemic antibiotics, a novel topical application of the taurine derivatives NCT and NBrT was suggested, because of their high tolerability and antiseptic activity including against biofilms without the development of resistance [18–20]. As expected, all clinical isolates were rapidly inactivated in time-kill assays *in vitro* by the active chlorine and bromine compounds used (Table 2).

**Table 2.** The susceptibility of the clinical isolates to antibiotics and haloamines

Bacterium	Resistant against*	Time for ≥3log_10_ reduction of c.f.u. ml^−1^ (min)†	
		1 % NCT (55 mM)	0.001 % BAT (32 µM) 0.02 % NBrT (1 mM)
* P. fluorescens *	Imipenem, meropenem	15	1
* S. lugdunensis *	None	15	1
* A. viridans *	None	>30‡	1
* B. pumilus *	Cefuroxime, fosfomycin, cefotaxime, cefepime	15	1
* K. marina *	None	30	1
* S. epidermidis *	Erythromycin	30	1
* S. capitis *	Penicillin, ampicillin	30	1

*Determined by agar diffusion tests according to European Committee on Antimicrobial Susceptibility Testing standards [24].

†Tests were done in 0.1M phosphate buffer at pH 7.1 and 37 °C; 1×10^6^ c.f.u. ml^−1^ were incubated in test solutions and quantitative cultures were assessed after inactivation of the antiseptics with 1 % methionine/1 % histidine.

‡2 log_10_ reduction after 30 min.

A regimen of one daily in-patient application of topical taurine derivatives was decided upon (Table 3, Fig. 1). The patient was handled according to the Declaration of Helsinki guidelines and gave written informed consent. Initially and for the first 3 days, the patient was treated for an hour with a gauze soaked in 1 % NCT. The NCT solution was put into a sterile spray bottle, releasing approximately 130 µl per single puff. The gauze was kept wet with the NCT solution by application of 3–5 puffs every 5 min. Treatment with NCT resulted in slow but effective regression of the lesions (Fig. 1c). On the fourth day, to further increase the therapeutic efficacy noticed with 1 % NCT as seen in herpes zoster and in a crural purulent wound [20, 21], it was decided to proceed with a sequential combination of NCT and NBrT at a concentration of 1 % each. The treatment was altered in that for half an hour a gauze soaked with 1 % NCT solution was used, followed by a gauze soaked with 1 % NBrT solution for half an hour. The combination treatment of NCT and NBrT evidently accelerated the therapeutic success as on the next day, i.e. the fifth day of treatment in total, the infection lesions turned soft and vastly regressed (Fig. 1d). At the end of the fifth day of treatment, the lesions were detached from the skin as they largely could be removed by simply rubbing the 1 % NBrT soaked gauze and without any severe bleeding (Fig. 1e). Since it would have been difficult for the patient to attend the clinic on a daily basis in the following period, it was decided to continue the topical treatment with NCT and NBrT at home and to augment it with a 0.1 % BAT solution, which shows higher stability than the taurine derivatives [22]. BAT solution was also prepared aseptically in distilled water and given to the patient in a spray bottle for daily use. The patient was advised to use two to three puffs (approximately 150 ml per puff), twice per day straight onto the scalp lesions. Visits at the outpatient department were planned every fourth day. At the next visit, i.e. the ninth day of treatment, the lesions continued to regress. The remaining lesions from the fifth day had almost disappeared, and the patient’s skin tissue had been markedly repaired (Fig. 1f). Progression of epithelialization continued as recorded on days 13 and 17 of treatment with no signs of recurrence (Fig. 1g, h) . Tissue repair and epithelialization were remarkable on day 25 of the treatment (Fig. 1i). Due to the evidently healed tissue, treatments with 1 % NCT and 1 % NBrT were halted, while 0.1 % BAT application was continued at home to avoid any chance of lesion recurrence. Thereafter, the patient was checked for lesion recurrence on a monthly basis. On day 60, the previously infected tissue had also returned to a normal colour (Fig. 1j). It was decided that the 0.1 % BAT application should be discontinued. The patient remained healed from the lesions during 4 months of observation after the treatment with the active halogen compounds.

**Table 3.** Treatment and progress of healing

Day	Treatment	Effect
1	1 % NCT 1 h gauze	Lesions detached
2–3	1 % NCT 1 h gauze	Lesions regressed slowly, but clearly
4–25	1 % NCT 0.5 h gauze 1 % NBrT 0.5 h gauze	Lesions turned soft, regressed quickly and were further detached (day 5)
5–60	1 % NCT 0.5 h gauze, 1 % NBrT 0.5 h gauze, plus 0.1 % BAT directly to the lesions	
9		Almost complete lesion regression, marked tissue repair
13,17		Progression of epithelialization
25	NCT + NBrT stopped	Tissue repair and healing complete
60	BAT stopped	Normal skin colour
90,120,150,180	No treatment	Healthy skin, no recurrence

## Discussion

### Medical importance of the bacteria isolated

The majority of studies investigating the clinical significance of bacterial synergy that may cause biofilms use data from experimental *in vitro* conditions to reflect clinical conditions [14, 29]. In this unique case of skin and subcutaneous infection with extended chronicity, the clinical microbiology investigation indicated a very interesting assortment of synergizing bacterial isolates. The scalp lesions were well presented and easily viewed macroscopically. However, to the best of our knowledge, there are no reports in the literature describing a multi-synergistic bacterial infection of the skin of this kind [11–16]. Multiple bacterial species were isolated from the overlaying infectious mass by swabs; therefore, they were presumptively associated with this skin and subcutaneous infection of the scalp. The isolates were mostly Gram-positive cocci apart from Gram negative rods of the *P. fluorescens* complex and Gram-positive rods of *B. pumilus *. It is known that in poly-microbial infections, a mixture of aerobic, anaerobic, Gram-positive and Gram-negative bacterial species synergize and confer metabolic advantages for co-existing growth [14]. Also, many of the species found readily produce biofilm (Table 1). It is true that these species may have been non-causative contaminants, but there is also a small but significant amount of evidence in the literature indicating their opportunistic virulence potential (Table 1). Strains of the *P. fluorescens* complex, although common inhabitants of soil and aquatic environments, recently have been considered of clinical significance due to the extent of their colonization in the lower gut and stomach microbiota in humans [30], and for being strong contributors to biofilm formation [31]. Their adaptation capacity to grow in diverse ecological niches, their alteration of virulence factor expression and, thus, their pathogenic behaviour due to their interaction with host elements explain their capability to produce a diversity of opportunistic infections [31]. Many strains of this complex are described as host invaders, especially during impairment of immune defence, and they are adequately described as human pathogens in various serious infections including wounds [31, 32]. *S. lugdunensis *, a coagulase-negative coccus, was also isolated from the tissue swabs. Strains of this species, although found regularly to be components of the normal skin flora and previously considered as non-pathogenic, recently have been implicated in a series of severe infections, particularly in soft tissue and skin [33]. Special adopted genetic machinery of this species is responsible for homophilic interaction between IsdC-containing molecules and neighbouring cells, contributing to the accumulation of biofilm *in vivo* [34]. *A. viridans *, also isolated from the tissue swabs, is a known opportunistic, pathogenic, Gram-positive coccus that does not produce catalase [35]. Importantly for our case, strains of this species contaminate hospital environments and room air. They gained clinical importance recently as proper identification distinguished them from other Gram-positive staphylococcal and streptococcal species [35]. Apart from serious systemic infections such as bacteraemia, endocarditis, meningitis and urinary tract infections, case studies of vasculitis and wound infections have been described [35, 36]. Until this report, to the best of our knowledge, no previous study indicated an involvement of *A. viridans* in biofilm-like lesions [14–16]. The clinically important *B. pumilus* strain was also isolated from the scalp tissue. Distinct from *Bacillus anthracis *, cutaneous infections clinically indistinguishable from anthrax but caused by *B. pumilus* have been described [37]. Cases of severe sepsis in infants and children who were otherwise immunocompetent patients suggest an occasional increased virulence of *B. pumilus* strains [38, 39]. The resistance to fosfomycin for our isolate had a medical impact (Table 2), because this antibiotic generally is a good choice since it penetrates into the biofilm and results in de-assembly of the biofilm structure [40]. Although most *Bacillus species* have the ability to produce biofilms in aquatic environments [41], only *Bacillus subtilis* is described as a sufficient biofilm former [42]. However, to our knowledge, none of the bacillus species has been implicated in biofilm-type infections so far, although their potential for involvement is alerted in the medical literature [8]. *Kocuria* micrococcal species inhabit the normal skin and mucous membranes of humans and animals. Although previously not considered as pathogenic, a recent rise in the incidence of both superficial infections and deep-seated or invasive infections is a warning [43]. An interesting case of bacterial sepsis by *K. marina* through skin breakdown is significant for the organism's capacity to contribute as a pathogenic microbe [44]. Although previous studies on clinical strains of *K. marina* showed a lack of ability to produce biofilms [43], other species such as *Kocuria kristinae* are active mucoid biofilm makers in dental implant placements [45]. Finally, amongst the coagulase-negative staphylococci, *S. epidermidis* and *S. capitis *, also isolated from our tissue swabs, are gaining clinical importance by causing increasing levels of bacteraemia and serious infections of newborns [46]. In particular, these two staphylococcal species have the capacity to produce biofilms and show resistance to antibiotics [47]. Therefore, they are considered as important contributors to this chronic scalp skin infection [47].

### Multi-bacterial skin infection of the scalp

As already mentioned, multi-bacterial species inherently form consortia for protection against environmental stress conditions [48]. Likewise, in the clinical environment, chronic infections may consist of synergizing microbial species [14, 15]. The most studied microbial biofilm-type infection that involves direct adhesion to host tissue is infective endocarditis. Microbial vegetations are formed by accumulations of bacteria, platelets and fibrin, which are adherent to the damaged epithelium of cardiac valves. However, *in vitro* and *in vivo* models employed do not provide adequate experimental conditions that reflect the true image of disease etiopathogenesis and therapy [9, 49]. The skin and soft subcutaneous tissues provide an anatomical region that is easier to investigate, diagnose and treat for biofilm infection. However, to investigate a chronic infection on the skin as a macroscopically easily viewed biofilm was not within the scope of this research study, due to the emergency of the clinical case requiring a swift and effective treatment. In the case described here, multiple pathogens may have synergized and caused a resistant chronic infection not previously described, which in many ways could have been a biofilm. Recently, the association between skin disease and biofilm infections has gained clinical importance as biofilms may be a serious cause of atopic dermatitis [50], the hidden cause of inflammatory ulcers usually diagnosed as pyoderma gangrenosum [27], and a hidden cause contributing to psoriasis pathogenesis [51].

Within biofilms, one bacterial species may concentrate with numerous synergizing species and form thick layers of exogenous tissue, as seen in this case [1, 11]. Although investigation of the biofilm-making capacity of each isolate was not in the scope of this study, it can be speculated for future clinical case emergence that the identified *P. fluorescens* and *Staphylococcus* spp. strains, which readily produce biofilm in human tissue and the clinical environment, optimized the conditions for the other non-biofilm forming species *A. viridans *, *K. marina* and *B. pumilus* to form this very well-organized thick infectious tissue overlaying the dermis (Fig. 1). This type of organized community of bacterial microcolonies resists radical elimination by antibiotic therapy [14, 16, 52]. Continuous recurrences during antibiotic treatment leave surgical removal as a sole therapeutic option, with no promise of success [52, 53]. This was also the sole therapeutic option remaining for the patient described here prior to attending our clinic. However, the pluralism of distinct microbial niches within poly-microbial infections makes the infection likely to persist, because the microbes can avoid killing by antibiotics [14, 15]. Usually, when antibiotics are used systematically, they only target a representative species mainly identified for a given systemic illness, letting other synergizing species live; thus, leading to the vast number of antimicrobial therapy failures [14, 16]. Resistance to the antimicrobial therapy used previously for the patient’s condition may be explained in this way, by the whole spectrum of antimicrobial resistance of all the isolates (Table 2), although no definite earlier diagnosis was ever made for our patient's infection.

Previously underestimated stress conditions, such as psychological induction and nutritional behaviour, further promote the formation of antibiotic-resistant biofilm-producing bacteria [54]. Under stress, the microbes take advantage of many kinds of host immune deficiencies, and the biofilm formed is either strengthened or acquires promoted virulence characteristics [54]. The higher age of our patient may also have been an important stress factor [55], allowing the infection to persist. In addition, the mild eosinophilia recorded may have paved the way for tissue invading infections, although this is controversial for bacteria [24].

The initial presumptive diagnosis for this patient was pyoderma gangrenosum, and the initial biopsy described multinucleated foreign body giant cells, which are known to develop in the course of immune reactions to foreign materials [27, 28]. It is likely that in the initial stages of the infection, pieces of the material that came into contact with the skin during the accident contained most of the bacteria that infected the patient. Primary histological examination may have identified the immune reaction of the skin, but not the contaminating microbes that formed this chronic biofilm-like infection. In this respect, the applied haloamines also may have counteracted immune responses that contributed to maintenance of the chronic infection [18, 28, 56].

### Successful treatment of the scalp infection with active halogen compounds

The efficiency of haloamines to treat this infection supports their future use as potential agents against multi-bacterial infections of the skin. The primary choice to use NCT in this at first unclear medical case was due to the resemblance of the lesions to infectious ones and to the knowledge that this taurine derivative kills biofilms [21, 57, 58]. There is broader experience of clinical application of NCT compared to bromamines. The later combination with NBrT was initiated to obtain a higher efficiency in the complete destruction of this biofilm-type infection, which was suggestive from two previous skin tissue infections, namely a purulent crural ulceration and a herpes zoster infection [18, 19]. Bromamines exert markedly higher reactivity than NCT against microbes in killing assays in buffer solution [59]. However, for active halogen compounds like chloramines and bromamines, it is the organic matter, such as the protein load, that directs their efficacy against infections [59]. In the presence of organic matter, the activity of NCT is generally increased and may override that of bromamines [59]. However, bromamines are surprisingly well tolerated by human cells, at least on the skin [22, 59], so that high concentrations can be used [60]. Broad clinical application of NBrT may be limited by its relatively low stability, so active bromine compounds like BAT are of interest, because they have a higher stability but similar properties [22]. It is conceivable from the therapeutic result that all three agents applied helped in the complete and rapid eradication of this noxious chronic infection in an elderly patient. None of these halogens proved toxic for the patient. This is in agreement with our previous experimental evidence for a low toxicity of halogens against human cells [59] and a remarkable tolerability by human subjects [20, 21, 61]. No relapse of infection occurred, as the patient remained free of symptoms for many months post-treatment.

In conclusion, this was a unique case of a chronic synergistic infection that was successfully treated with the active halogen compounds NCT, NBrT and BAT. This case marks their potential for clinical use in similar cases. The multi-bacterial infection was composed of an assembly of partly unusual pathogens and not by predominantly biofilm-making virulent bacteria like *S. aureus* and *Pseudomonas aeruginosa* [62]. However, these bacteria may have become life-threatening for the elderly patient if they had crossed the dermal barrier.
